# Shaping ability of three nickel-titanium rotary instruments in simulated L-shaped canals: OneShape, Hero Shaper, and Revo-S

**DOI:** 10.1186/s12903-021-01734-6

**Published:** 2021-07-26

**Authors:** Pouyan Vakili-Gilani, Saeid Tavanafar, Abdul Rahman Mohammad Saleh, Hamideh Karimpour

**Affiliations:** 1 Department of Clinical Sciences, Ajman University, Ajman, United Arab Emirates; 2 Department of Oral and Maxillofacial Surgery, School of Dentistry, Shiraz University of Medical Sciences, Shiraz, Iran; 3 General Practitioner, Private Practice, Shiraz, Iran

**Keywords:** Hero Shaper, Nickel-titanium, OneShape, Revo-S, Shaping ability, Simulated canals, Rotary

## Abstract

**Background:**

Shaping ability of a file plays an important role during instrumentation in an endodontic treatment. This study aimed to compare the shaping ability of OneShape (OS), Hero Shaper (HS), and Revo-S (RS) instruments in simulated L-shaped canals.

**Methods:**

Forty-eight simulated L-shaped canals were prepared to an apical size of 25 using OS, HS, and RS (all from Micro-Mega SA, Besançon, France), (n = 16 canals/group) systems. The amount of resin removed after each canal's preparation was measured and compared after producing a composite image made from the superimposition of pre and post-instrumented canals. Canal aberrations and the preparation time were also recorded. The data were statistically analysed by using ANOVA, Tukey, and Chi-square tests.

**Results:**

One file fractured during instrumentation in the RS group. A significant difference was found at the apical end of the prepared simulated canal between the groups, with RS showing the least amount of resin removal from the inner side of the canals and HS showing the highest amount of resin removal from the outer side (*P* < 0.05). Regarding the total width of the canals after preparation, a significant difference was found between the groups at the apical end and the straight portion of the canals, and RS removed the least amount of resin at the straight portion of the canals (*P* < 0.05). No statistically significant differences were found between the different instruments regarding canal aberrations' incidence (*P* > 0.05).

**Conclusions:**

All of the files showed a tendency to straighten the canals, whereas OS files maintained the original canal curvatures well.

## Background

The instrumentation aims to shape the canals to facilitate cleaning and obturation, preventing disease progression and promoting healing. Rotary NiTi (nickel-titanium) instruments are designed to shape the canals. Their performance and safety have always been a subject of interest among practitioners in the presence of anatomical challenges. Canal curvature is a parameter that can challenge the performance of a file; hence respecting the anatomy of a canal in the presence of curvature is a desired characteristic of any rotary instrument considering the root canal morphology [1, 2].

The OneShape (OS; Micro Méga, Besançon, France) is a single-file canal preparation system made of conventional NiTi alloy that works on full clock rotation. This system was introduced to the market after two reciprocating single-file systems named WaveOne (Dentsply Maillefer, Ballaigues, Switzerland) and Reciproc (VDW, Munich, Germany) promising results. The OS file has a tip size of 25 and a constant taper of 0.06 with different cross-sections along its length, changing from S-shaped to concave triangular shape near the tip. This system requires only one file working in a clockwise rotation to prepare the canals up to the apical size of 25. These features have decreased the preparation time by OS compared to other single and full sequence file systems [3, 4]. Bürklein et al. found that OS required less time in comparison with another single file and a full sequence system to prepare canals in extracted teeth although the clinical significance this difference is questionable [3]. The file has features such as Anti Breakage Control (ABC) and asymmetric file design, which is claimed by the manufacture to increase the safety of the system [5].

Hero Shaper (HS; Micro Méga, Besançon, France) is a full-sequence system introduced as a modification of Hero 642 in terms of helix pitch and helix angle with a shortened handle. HS files have a positive rake angle, large inner core, and ABC, incorporated into the design to increase the files' efficacy and safety. The system consists of six files with tip sizes of 20, 25, and 30 and is grouped in tapers of 0.4 and 0.6. The manufacturer suggests three protocols, namely yellow, red, and blue, based on the canal’s anatomy to be instrumented. A sufficient amount of studies are available on this system to make it a proper baseline for the evaluation of rotary instruments [6–8].

Revo-S (RS; Micro-Mega SA, Besançon, France) is another full-sequence system introduced after HS by the same company, which consists of three files with a constant apical size of 25. The main feature of this system is the asymmetry in the cross-section or offset mass of rotation [9]. This feature is claimed to enhance the negotiation of curved canals due to the files' increased flexibility [10]. The manufacturer also claims that the file's helical design facilitates debris movement away from the apex because of the increased available volume of space between the file and the canal's surface. This asymmetric design was not incorporated in Revo-SC2. Claimed by the manufacture, this file's asymmetry balances the forces and ensures the instrument's guidance up to the apical region. Like HS, this system also benefits from variable pitch angle.

Although HS and RS might be considered traditional systems compared to the latest rotary instruments, especially the new wave of single-file systems. These are the products of constant changes and improvements in NiTi instruments' designs over a short period by the same manufacturer. Evaluation of these systems' behaviour may give us some insights into the effect of changes on our understanding of different designs and sequences of rotary files on their shaping ability. Therefore, this study aimed to evaluate three NiTi files' shaping ability regarding their centricity in simulated L-shaped canals.

## Methods

### Sample size

Sample size was calculated with assumption of alpha = 0.05, power = 80% maximum difference of means (d) = 0.05 and pooled standard deviation (s) = 0.04 (4) and 3 levels(k = 3) by this formula $$n* = \frac{{2{s^2}{{({z_{1 - {\raise0.7ex\hbox{$\alpha $} \!\mathord{\left/ {\vphantom {\alpha 2}}\right.\kern-\nulldelimiterspace}\!\lower0.7ex\hbox{$2$}}}} + {z_{1 - \beta }})}^2}}}{{{{(d)}^2}}},n = \sqrt {k - 1}$$. So the sample size in each group approximately was 16.

### Simulated canals

Forty-eight simulated L-shaped canals were used in this study (Endo Training Block-S; Dentsply Maillefer, Ballaigues, Switzerland, with 0.02 taper, 0.15 mm apical diameter, 17 mm length, and 40 curvature). The patency of the canals was confirmed by passing a size 10 K-file (Micro Méga, Besançon, France) just beyond the apex, and the unity in the angles and length of the curvatures were confirmed before distribution of the blocks by taking pictures of the samples on a photography stand. After assuring that the samples are standard, they were randomly divided into three groups (n = 16 canals/group) and were numbered.

**Fig. 1:**
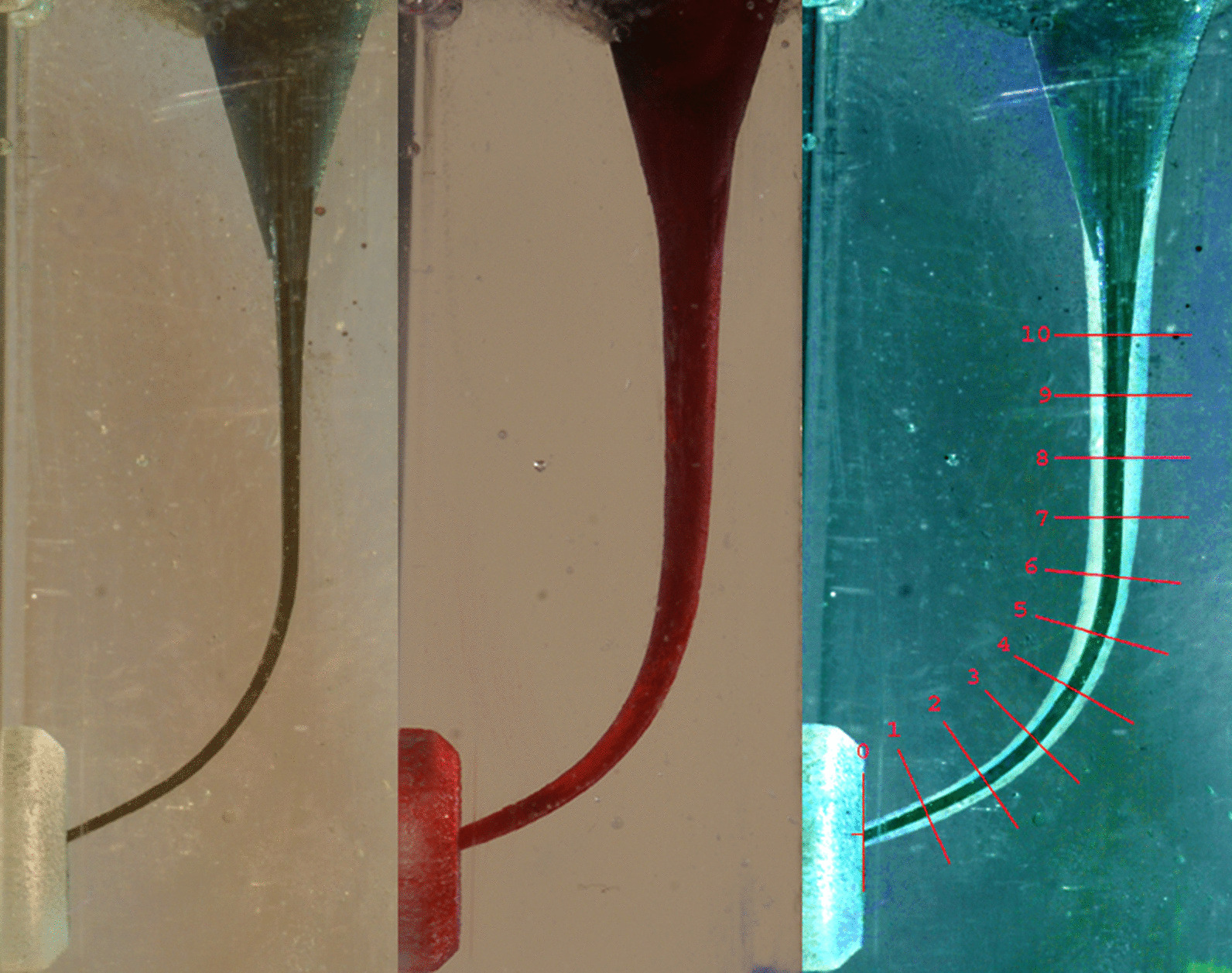
Pre-and post-instrumentation images, and superimposed template on the composite image

All canals were injected with black ink (Parker Quink, Parker, France) to obtain a clear pre-instrumentation image (Fig. 1). The canals were photographed using a digital camera (Sony Alpha DSLR-A100 camera with DSLR-A100 macro lens, Sony, Japan) on a fixed stand with constant settings. All the canals were rinsed with saline before and after instrumentation prior to ink injection.

### Instrumentation of L-shaped canals

A new instrument was used for each canal in all groups. Glyde-Prep (Dentsply Maillefer, Ballaigues, Switzerland) was used as a lubricant before using each instrument, and saline was used for irrigation during preparation. The canals were instrumented using the protocols suggested by the manufacturer described in the following sections without glide path preparation or additional use of hand files except for recapitulation with a size 10 K-file.

#### Group A

The OS file (tip size, 25; apical taper, 0.06) was used in a full clockwise rotation generated by an X-Smart motor (Dentsply Maillefer, Ballaigues, Switzerland), and the speed and torque were adjusted to 400 rpm and 4 Ncm. The files were used in a slight pecking motion according to the manufacturer’s instructions. The flutes of the instrument were cleaned after each retrieval of the file from the simulated canal.

#### Group B

The HS files were used following the yellow sequence with file size 25 taper 0.6 used as a modification to the protocol to achieve the same apical size and taper of prepared canals as that of other groups. According to the manufacturer's torque guide, the motor was set at the speed of 400rmp with the torque of 1 to 2 set for each instrument. The instrument sequence was as follows:

1. A 0.06 taper size 20 instrument for 2/3 of the WL.

2. A 0.04 taper size 20 instrument for the full WL.

3 A 0.04 taper size 25 instrument for the full WL.

4 A 0.06 taper size 25 instrument for the full WL.

#### Group C

The RS files were used up to the size 25 and taper of 0.06 in a full clockwise rotation with a rotational speed of 400 rpm generated by the X-Smart motor (Dentsply Maillefer, Ballaigues, Switzerland), and the torque was adjusted to 2 Ncm. The files were used in a slight pecking motion according to the manufacturer’s instructions. The instrument sequence was as follows:

1. A 0.06 taper size 25 instrument (SC1) 2/3 of the WL.

2. A 0.04 taper size 25 instrument (SC2) for the full WL.

3. A 0.06 taper size 25 instrument (SU) for the full WL.

An endodontist with more than 20 years of experience and the history of conducting studies on resin blocks prepared all canals, and a total of 48 L-shaped canals were prepared [4, 11]. Canals were irrigated during preparation by using saline. A new instrument was used to prepare four canals only, and the flutes of all instruments were cleaned after retrieval of the instruments from the canals during instrumentation or after three pecks.

### Image analysis and assessment of canal preparation

The post-instrumented canals were subsequently filled with red ink (Parker Quink, Parker, France) and were photographed again under identical conditions to the pre-instrumentation images. The pre-and post-instrumentation images were superimposed into a composite image using a computer software program (Adobe Photoshop Elements 7.0, Adobe Systems Incorporated, San Jose, CA, USA). The post-instrumentation image was faded, inverted (converted to a negative photo), and superimposed in Photoshop for composite image production. A measuring template was also superimposed on the composite images (Fig. 1). The amount of resin removal due to instrumentation was measured using ImageJ 1.46r software (Wayne Rasband, National Institutes of Health, USA) perpendicularly to the surface of the canal at 22 measuring points (11 on each side of the canal). The measurement points (MP) were arranged in 1-mm steps: points 0 corresponded to the canal's endpoint, 7 to the beginning of the curve, and points 7 to 10 belonged to the straight portion of the canal. A second examiner who was blinded to all experimental groups carried out the canal shapes' assessments before and after instrumentation.

Centring ability was assessed for each measuring point by analyzing the amount of resin removed at the inner side versus the outer side using paired *t* test (*p* < 0.05). Canal preparation with no significant differences between the amounts of resin removed from the inner and outer side of a canal was considered as good centring ability. 

The canal preparation time, which included total active instrumentation, cleaning of the instruments' flutes, and irrigation, was recorded. The amount of time spent to change the files or adjust stoppers was excluded to facilitate the files' efficacy. Canal aberrations were determined by two clinicians blinded to the canal preparation instruments by using composite images. Assessments were performed based on an apical zip, narrowing, ledge, and the danger zone. The canal aberrations were defined according to Ersev et al.[7]

### Statistical analysis

Statistical evaluations were performed with SPSS software (IBM SPSS Statistics 21, SPSS Inc., Chicago, USA). The normality of the data was verified for each set of measurements by using the Kolmogorov–Smirnov test. The results were statistically.

analysed using one-way analysis of variance (ANOVA) and the post hoc Tukey test. ANOVA and the post hoc Tukey were also used to analyse the preparation times, and the Chi-square test was used to analyse the incidence of canal aberrations. The significance level was set at *P* < 0.05.

## Results

Only one SC2 file in the RS group fractured during instrumentation. Consequently, the sample was substituted with a new one, and another set of instruments were used. No sign of deformation was noticed visually on RS and HS files, but all the OS files showed deformity signs.

**Fig. 2:**
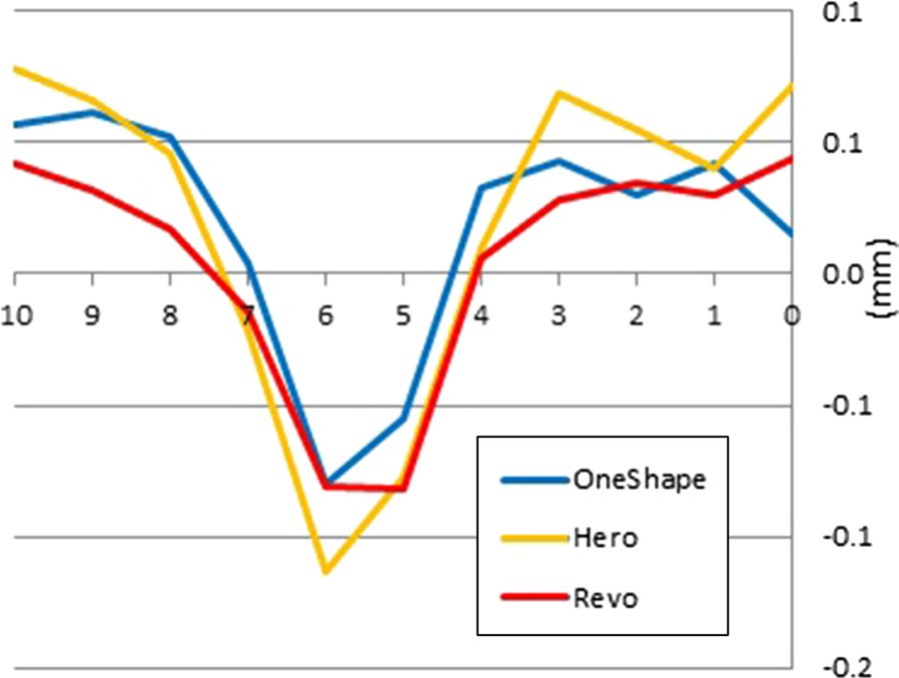
Direction and the amount of canal transportation (mm) at different measurement points

A significant difference was found at the apical end of the prepared simulated canal (MP 0) between the groups, with RS showing the least amount of resin removal from the inner side of the canals and HS showing the highest amount of resin removal from the outer side (*P* < 0.05; Table 1). RS removed the highest amount of resin from the inner side of the canals at the endpoint of curvature, however not significantly different from the other groups. HS showed the least amount of resin removal from the canals' outer surface at the curvature's apex but the highest amount of resin removal from the outer side at the beginning of the canals (*P* < 0.05, Table 1). RS removed significantly less resin from the outer side at the canals' straight portion (*P* < 0.05, Table 1). OS showed the highest centricity at the terminal portion of the canals and the curvature's beginning, followed by RS. This pattern was reversed at the other points of canals by RS, showing a higher centricity than OS (Fig. 2).

**Table 1:** Means of removed resin (mm) and standard deviations (SD at the different measurement points after root canal preparation

	Inner Canal Wall (mm from the apex)											Outer Canal Wall (mm from the apex)										
	0	1	2	3	4	5	6	7	8	9	10	0	1	2	3	4	5	6	7	8	9	10
**OS**																						
Mean	0.07	0.08	0.10	0.11	0.15	0.22	0.25	0.23	0.22	0.22	0.21	0.09	0.12	0.13	0.16	0.18	0.16	0.17	0.23	0.27	0.28	0.27
SD	0.02	0.02	0.02	0.02	0.02	0.03	0.02	0.02	0.02	0.02	0.02	0.03	0.02	0.02	0.02	0.02	0.02	0.02	0.03	0.03	0.04	0.03
**HS**																						
Mean	0.06	0.08	0.10	0.11	0.15	0.23	0.26	0.24	0.23	0.22	0.21	0.12	0.12	0.13	0.16	0.16	0.14	0.16	0.20	0.26	0.28	0.29
SD	0.02	0.03	0.03	0.03	0.03	0.02	0.02	0.03	0.03	0.03	0.03	0.04	0.03	0.04	0.02	0.02	0.03	0.02	0.02	0.02	0.03	0.04
**RS**																						
Mean	0.05	0.08	0.10	0.13	0.16	0.22	0.24	0.23	0.22	0.22	0.20	0.09	0.11	0.13	0.16	0.17	0.14	0.16	0.20	0.24	0.25	0.24
SD	0.02	0.02	0.02	0.02	0.03	0.03	0.03	0.02	0.02	0.01	0.02	0.03	0.02	0.02	0.02	0.02	0.02	0.03	0.03	0.03	0.03	0.03
	*											*				*			*	*	*	**

^*^P < 0.05; **P < 0.01; (ANOVA and post hoc Tukey test)

**Fig. 3:**
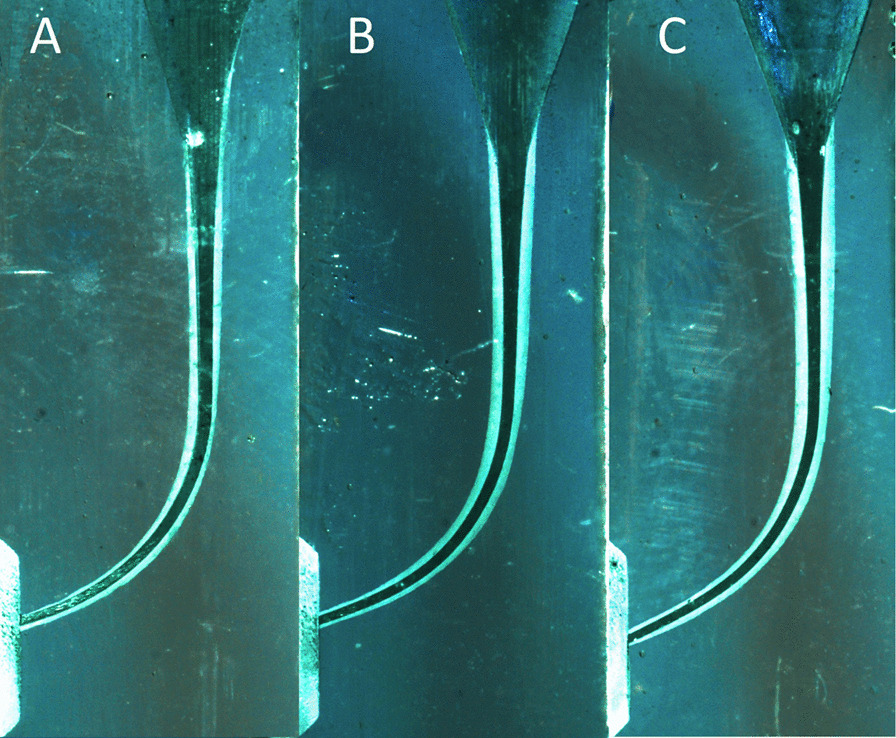
Representative images of the simulated canals instrumented using (**A**) HS, (**B**) RS, (**C**) OS

Regarding the total width of the canals after preparation, a significant difference was found between the groups at the apical end and the straight portion of the canals only, with RS removing the least amount of resin at the straight portion of the canals (*P* < 0.05; Table 2).

**Table 2:** Means of canal width after instrumentation (mm) and standard deviations (SD at the different measurement points after root canal preparation

	Measurement points (mm from the apex)										
	0	1	2	3	4	5	6	7	8	9	10
**OS**											
Mean	0.31	0.37	0.42	0.47	0.54	0.59	0.65	0.71	0.76	0.82	0.88
SD	0.04	0.02	0.02	0.03	0.03	0.02	0.02	0.02	0.02	0.02	0.02
**HS**											
Mean	0.34	0.38	0.42	0.49	0.52	0.60	0.66	0.73	0.78	0.83	0.89
SD	0.04	0.04	0.04	0.03	0.03	0.03	0.02	0.02	0.02	0.02	0.02
**RS**											
Mean	0.31	0.37	0.43	0.49	0.54	0.59	0.63	0.69	0.74	0.79	0.85
SD	0.03*	0.03	0.03	0.03	0.05	0.04	0.04*	0.04**	0.03***	0.04**	0.03**

^*^P < 0.05; **P < 0.01; ***P < 0.001(ANOVA and post-hoc Tukey test)

### Canal aberrations

The results of the canal aberrations are presented in Table 3. No statistically significant differences were found between the different instruments regarding canal aberrations' incidence (Fig. 2, Chi-square test, *P* > 0.05).

**Table 3:** Incidence of aberrations

	OS	HS	RS
Ledge	0	2	1
Danger Zone	0	0	0
Narrowing	0	1	0
Zip and Elbow	1	3	1

Chi-square test, no significant difference (*P* > 0.05)

### Centring ability

OS showed the highest centricity along the simulated canals, followed by RS (Fig. 1). HS tends to cause more straightening by removing the higher amount of resin from the outer than inner side of the canals at the apical part. The least centricity was also noticed by the same file at the beginning of the curvature (MP 5–6), shown in Fig. 2.

### Preparation time

Considering the preparation time, a significant difference (*P* < 0.05) was found between OS (63.7 s) and HS only (73.6 s); no significant difference was found between RS (68.8 s) and the other two files (*P* < 0.05) (Fig. 3).

### Discussion

This study aimed to compare OS single file system's shaping ability with RS and HS. RS and HS were used in this study as they have some common features in design with OS because all these three systems are products of the same company. They also demonstrate a gradual change from a full sequence system to a single file system which is OS.

Various methods have been used to evaluate the shaping ability of rotary or manual files [12–19]. Methods vary based on the parameters being evaluated and the devices used to measure changes. Studies can be performed either on natural teeth or simulated canals. Studies on natural teeth can be conducted by using images of sectioned teeth, dental x-rays, cone beam radiography and micro-computed tomographic imaging [12–15]. Simulated canals are being used as an alternative to natural teeth when comparing the shaping ability of different files because of their advantages [4, 16–19]. Simulated canals are standard in canal curvature, length, diameter, and hardness. Also, it is easy to measure changes on them after instrumentation [20, 21]. Therefore, simulated L-shaped canals were used in this study due to the importance of standardization of experimental setting, minimizing the possible variables and ease of reliably obtaining pre and post-instrumentation images in accordance with previous studies [4, 11].

The simulated L-shaped canals in this study were prepared up to the size of 25 to make the comparison between the files easier because OS when used as a single file, prepares canals to the minimum size of 25. The HS system's yellow sequence was modified using the file 25 taper 0.06 instead of the file 30 taper 0.04 to reach a closer prepared canal shape compared to the other two systems.

### Amount of resin removed

More centricity with OS at the apex may be due to the fact that only one file was passed to the canal's apex. However, in the other two systems, the apex was instrumented twice with files of the same size but different tapers to achieve the final shape. This could attribute to the more amount of resin removal from the outer side of the canals at the terminal point of the canals. Consequently, the increased number of passes and preparation time in RS and HS full sequence systems can cause eccentricity at the apical point [22]. Furthermore, OS has a variable cross-section with a small inner core in its middle portion and an S-shaped cross-section in its coronal portion. These features may contribute to higher flexibility and more centricity [23, 24]. The offset mass of rotation causing swaggering motion in RS could be the reason for higher maintenance of canal curvature and less mount of resin removal from the outer canal wall in comparison with HS group [18].

### File separation and canal aberrations

Only one SC2 rotary file fractured. All of the instruments prepared the simulated canals to the full length. Each rotary file sequence is used to prepare only one canal. HS created two ledges, three zip and elbows, and a narrowing effect. However, the higher number of aberrations in the HS group was not statistically significant compared to other groups. A similar result regarding zip formation using HS was reported by Perez et al. but two other studies reported fewer canal aberrations [6, 8, 25]. Because all three systems have non-cutting tips and are made of conventional NiTi alloy, higher canal aberrations may be explained due to the differences in design, flexibility, and the number of instruments used among the three systems [22, 24]. HS has a larger core, hence less flexibility in comparison to OS and RS [23]. Unlike HS, RS has an offset mass of rotation and OS has a variable cross-section that increases its flexibility. Offset mass of rotation which is present in RS has been associated with the maintenance of canal curvature [18]. Another possible reason for the observation of higher canal aberration in the HS group can be due to the modification in sequence of the HS system in this study.

Care must be exercised while extrapolating the results of the studies conducted on resin blocks. Resin blocks are softer than natural teeth, and the debris formed during instrumentation is not that of a natural tooth. Therefore, the files might not be as safe and efficient during natural tooth preparation [20]. It is also important to remember that the root canals in natural teeth are not merely a single canal as presented in simulated resin blocks. A root canal system is more complicated [26]. In addition, the modification on sequencing HS files and its potential effect on the outcome of this study needs to be considered and emphasized. The use of ratios instead of actual measurements while evaluating centricity may eliminate the need for modifications. More studies are required to evaluate and assess the effect of the number of instruments used during instrumentation on maintaining the geometry of the canals, especially in the apical portion. The effect of the operator’s experience and the performance of single-file systems in comparison with full sequence systems also requires to be examined.

## Conclusions

Within this study's parameters, all of the files showed a tendency to straighten the canals, whereas OS files maintained the original canal curvatures well. Single files that are less tapered should be preferred when preparing severely curved canals to maintain the original canal curvatures.

## Data Availability

The datasets used and/or analysed during the current study available from the corresponding author on reasonable request.

## Abbreviations

OS: OneShape
HS: Hero Shaper
RS: Revo-s
ANOVA: Analysis of Variance
Niti: Nickel–titanium
ABC: Anti Breakage Control
WL: Working length
MP: Measurement points
